# Virome of the Russian Grapevine Germplasm: A Final Study and Summary

**DOI:** 10.3390/plants15172698

**Published:** 2026-09-02

**Authors:** Daria Belkina, Daria Karpova, Elena Porotikova, Elizaveta Shevchenko, Svetlana Matviets, Natalya Brusnova, Svetlana Vinogradova

**Affiliations:** 1 Skryabin Institute of Bioengineering, Research Center of Biotechnology of the Russian Academy of Sciences, Leninsky Prospect, 33, Build. 2, 119071 Moscow, Russia; 2 North Caucasian Federal Scientific Center of Horticulture, Viticulture, Wine-Making, 40 Years of Victory Street, Build. 39, 350901 Krasnodar, Russia

**Keywords:** *Vitis vinifera*, grapevine virome, RNA-Seq, grapevine viruses, grapevine germplasm, high-throughput sequencing, genetic diversity, phylogenetic analysis

## Abstract

Ampelographic collections play an important role in the conservation of grapevine genetic resources and therefore require continuous phytosanitary monitoring. In this study, the virome of grapevines from the Magarach ampelographic collection in Russia was analyzed using total RNA high-throughput sequencing. A total of twenty-seven grapevine viruses and four viroids were identified. Two viruses were characterized as putative novel species: (+) ssRNA grapevine umbra-like virus 5 (GULV-5) and the bipartite (+) ssRNA grapevine Magarach secovirus (GMSV), which, together with related viruses, may represent a novel genus within the family *Secoviridae*. Among the economically important viruses, the most prevalent were grapevine fanleaf virus (76%), grapevine leafroll-associated virus 1 (39%), and grapevine virus A (33%). Mixed infections involving two or three of these viruses were detected in 50% of the analyzed grapevines. Grapevine virus D was detected in Russia for the first time. Phylogenetic analysis of 222 assembled virus and viroid genome sequences revealed high genetic diversity. The obtained results were summarized and compared with previous virome studies conducted on four Russian ampelographic collections.

## 1. Introduction

Grapevine is an important agricultural crop that is grown all over the world and is a significant part of the economy of many countries. It is used for the production of wine and other alcoholic beverages, as well as table grapes, grape juice, raisins and other processed products [1]. Like other agricultural sectors, viticulture is currently facing new challenges associated with climate change [2,3,4]. In recent years, extreme climatic events have had a serious impact on vineyards worldwide, negatively affecting grape yield and wine production [5]. Ongoing climatic changes and the expansion of viticulture into new growing regions make it necessary to apply the full diversity of genetic resources to the development of varieties and hybrids with improved resistance to biotic and abiotic stresses [6,7].

Ampelographic collections play an important role in the conservation of grapevine genetic resources [8]. Most studies of ampelographic collections focus on the characterization of varieties, including the assessment of ampelographic traits, genetic diversity, and resistance to biotic and abiotic stresses [9,10,11,12]. One of the challenges in maintaining genetic collections is the occurrence of pathogens, including viruses, which can persist and spread within vineyards over time. Viral infections can reduce the agricultural and breeding value of varieties and weaken plants, increasing their sensitivity to biotic and abiotic stresses [13]. To date, more than a hundred grapevine viruses are known, some of which are associated with severe economic losses [14]. Previous studies of grapevine genetic collections for viral infections have been conducted in several countries: Portugal, Romania, Croatia, and Algeria [15,16,17,18]. Using ELISA and PCR-based methods, these studies analyzed the distribution of economically important viruses, including grapevine fanleaf virus (GFLV; *Nepovirus foliumflabelli*), grapevine leafroll-associated virus 1 (GLRaV-1; *Ampelovirus univitis*), GLRaV-2 (*Closterovirus vitis*), GLRaV-3 (*Ampelovirus trivitis*), and grapevine virus A (GVA; *Vitivirus alphavitis*). In addition, grapevine fleck virus (GFkV; *Maculavirus vitis*), GLRaV-4 (*Ampelovirus tetravitis*), arabis mosaic virus (ArMV; *Nepovirus arabis*), and grapevine virus B (GVB; *Vitivirus betavitis*) were also detected. Several studies have focused on the distribution of the recently discovered grapevine red blotch virus (GRBV; *Grablovirus vitis*) in ampelographic collections in Switzerland, France, the USA, and Italy [19,20,21]. Reynard et al. used not only RT-PCR but also high-throughput sequencing (HTS) for GRBV detection. All GRBV-infected plants were destroyed [19].

In our previous studies, four Russian ampelographic collections were analyzed using total RNA sequencing. These studies included the Anapa ampelographic collection in the Krasnodar region [22], two ampelographic collections in Dagestan [23], and the Don ampelographic collection, named after Ya. I. Potapenko, in the Rostov region [24]. The analyses revealed the presence of both economically important grapevine viruses and viruses that had not previously been reported in Russian vineyards. In addition, seven novel grapevine viruses were discovered, including dsDNA grapevine pararetrovirus (GPRV, *Caulimoviridae*), bipartite dsRNA grapevine alphapartitivirus (GAPV, *Partitiviridae*), bipartite (+) ssRNA grapevine secovirus (GSV, *Secoviridae*), and four (+) ssRNA grapevine umbra-like viruses (GULV-1, GULV-2, GULV-3, GULV-4, *Tombusviridae*).

The aim of the present study was to investigate the virome of the Magarach ampelographic collection, one of the oldest and largest grapevine collections in the world that includes varieties originating from diverse viticultural regions of Europe, Asia, Africa, and North America [25]. The collection contains 4120 accessions, including 1432 local and indigenous varieties, as well as 763 accessions from the special breeding collection of the All-Russian National Research Institute of Viticulture and Winemaking “Magarach” [26]. It includes table, seedless table, wine, dual-purpose, and rootstock varieties [27]. The passport database of varieties of the Magarach ampelographic collection is included in the Vitis International Variety Catalogue (VIVC) [28]. Due to the uniqueness and high genetic value of this collection, phytosanitary monitoring, assessment of virus and viroid distribution, and analysis of their genetic diversity are essential for the conservation of grapevine genetic resources.

This article presents the results of virome analysis of grapevines from the Magarach ampelographic collection and provides a summary review of the viromes of the Russian grapevine germplasm collections.

## 2. Results

### 2.1. RNA-Seq Data Analysis

Sequencing of 51 libraries generated 821 million raw reads, with an average of 17.9 million reads per library (Table S1). After removal of low-quality and duplicate sequences, 106 million reads remained, corresponding to an average of 5.3 million reads per library. Preprocessed reads were assembled into contigs using the Geneious and SPAdes assemblers. The Geneious assembler generated an average of 72,603 contigs per library with N50 = 726 bp. SPAdes produced an average of 4639 contigs per library with N50 = 1379 bp (Table S1).

### 2.2. Identification and Phylogenetic Analysis of Known Grapevine Viruses and Viroids

A total of 27 virus species, including two putative novel species, and four viroid species were identified in grapevines from the Magarach ampelographic collection (Tables S2 and S3). The detected viruses and viroids belonged to 17 genera (Table 1).

**Figure 1:**
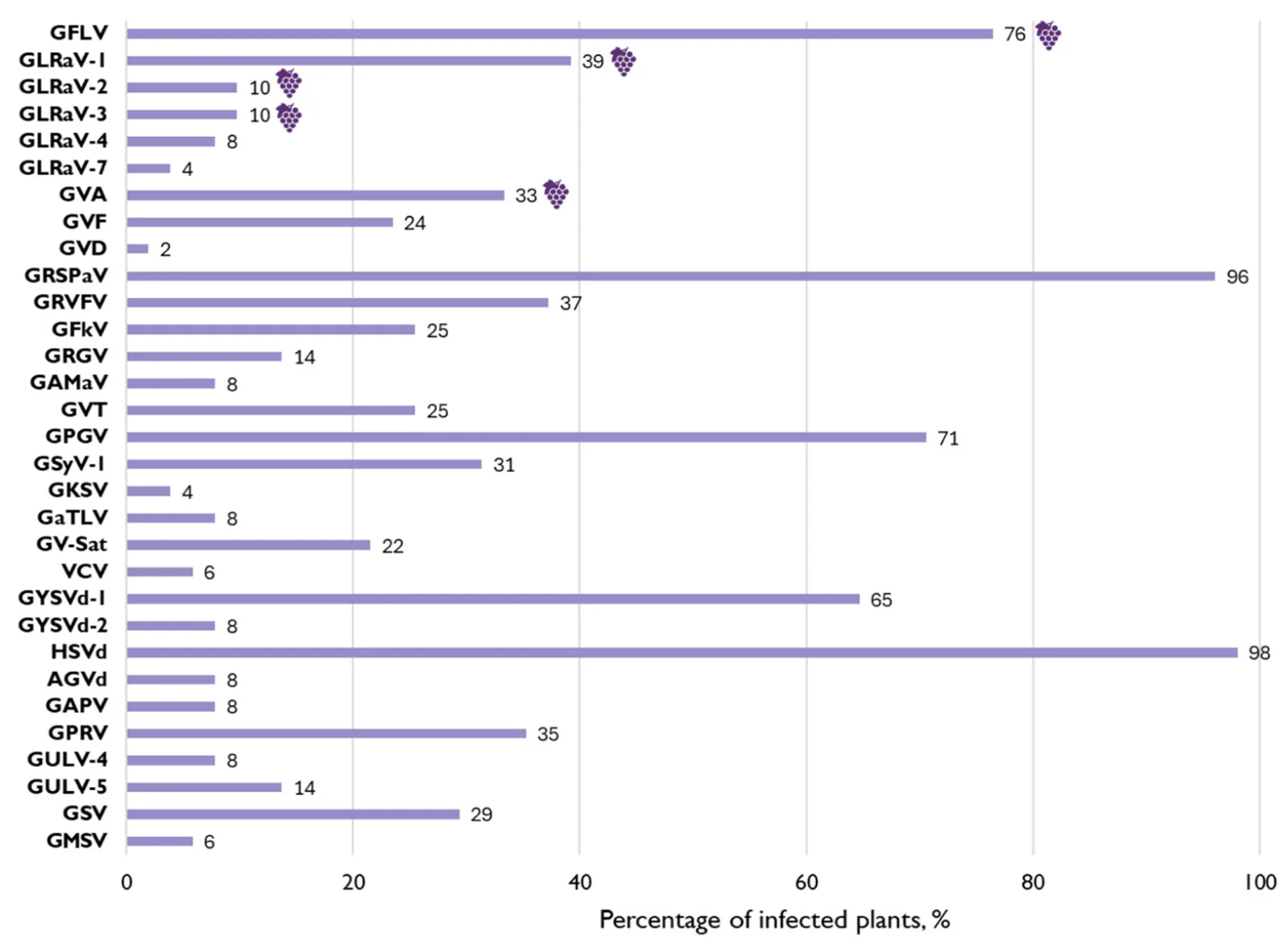
Distribution of viruses and viroids in grapevines from the Magarach ampelographic collection (percentage of the total number of samples). Economically important viruses are shown with a 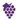.

The most prevalent viruses and viroids in grapevines from the Magarach ampelographic collection were GRSPaV (detected in 96% of analyzed vines), GFLV (76%), GPGV (71%), and HSVd (98%) (Figure 1). In addition to GFLV, four economically important viruses were detected: GLRaV-1 (39%), GVA (33%), GLRaV-2 (10%), and GLRaV-3 (10%).

Mixed infections involving two to seventeen viruses were detected in 50 of the analyzed grapevines (Table S1). Fifteen grapevines were simultaneously infected with the economically important viruses GVA, GLRaV-1, and GFLV, while two additional vines were co-infected with GLRaV-2, GLRaV-3, and GFLV. Nine additional grapevines were infected with two viruses from the above group in different combinations.

TblastX analysis of contigs and mapping of reads to the reference viral genomes enabled the assembly of 201 virus and viroid genome sequences, as well as 11 coat protein (CP) gene sequences of GFkV. In some cases, grapevines were simultaneously infected with several genetic variants of the same virus or viroid. For example, the presence of whole-genome contigs enabled separation of distinct isolates of GFLV, GRSPaV, GVA and other viruses. In contrast, insufficient coverage combined with high sequence polymorphism prevented reliable assembly of GLRaV-1 genomes.

The assembled virus and viroid sequences were included in a phylogenetic analysis together with representative members of phylogroups or all isolates available in GenBank (Table S4).

Phylogenetic analysis of GFLV included the complete global diversity of RNA1 and RNA2 sequences available in GenBank, together with 38 RNA1 and 38 RNA2 sequences assembled in this study. Mixed infections with multiple GFLV genetic variants were detected in six samples containing two variants and in two samples containing three variants. In addition, GFLV satellite RNA (satRNA) was detected in five samples (Table S2). Phylogenetic analysis showed that the RNA1 sequences formed two large clades (Figure S1). The first clade consisted mainly of isolates from Dagestan (Russia) and also included 15 Magarach isolates, 10 of which formed a distinct subcluster. The second large clade contained isolates from Europe, Africa and North America and was subdivided into many groups of varying sizes. This clade included 22 Magarach isolates. Three groups within this clade consisted exclusively of French isolates. A small intermediate cluster located between the two major clades included four Asian isolates and one isolate from the present study. 

Clustering of GFLV RNA2 sequences was generally consistent with the RNA1 phylogeny (Figure S2). However, several Asian isolates from China, Japan, and Iran clustered together with the Dagestan and Magarach isolates. Of the 38 Magarach isolates, 18 belonged to this clade. The remaining 20 clustered within the second clade formed by isolates from Europe, Africa, North America, and Western Asia. Interestingly, Magarach isolates detected on the same plant mostly belonged to different clades.

Phylogenetic analysis of viruses associated with grapevine leafroll disease showed that, among the four Magarach GLRaV-2 isolates, two belonged to phylogroup PN, while one isolate each belonged to phylogroups H4 and PV20 (Figure S3) [29,30]. The Magarach GLRaV-3 isolates together with previously published Russian isolates clustered within phylogroups I and II, which were the most prevalent groups in this study (Figure S4) [31,32,33]. One Magarach isolate clustered together with the GLRaV-3m strain. Of the three assembled GLRaV-4 genomes, one belonged to strain 5, which was previously reported in Russia (Figure S5). The other two isolates clustered together with GLRaV-4 Ob [34]. The GLRaV-7 isolate identified in this study was phylogenetically close to another Russian isolate previously detected in the Dagestan ampelographic collection (Figure S6).

Phylogenetic analysis of the complete Russian GPGV genomes, including isolates detected in wild *V. vinifera* ssp. *sylvestris* from protected natural areas, demonstrated high sequence identity with each other and closeness to the European isolates (Figure S7). The Magarach isolates did not form separate phylogroups. Additional phylogenetic analysis of the MP/CP region indicated clustering of the Magarach isolates together with latent GPGV strains (Figure S8) [35,36,37]. Multiple sequence alignment of this region confirmed the absence of an early stop codon at the 3’ end of the MP-encoding ORF in the Magarach isolates (Figure S8). 

The Magarach GRSPaV isolates belonged to phylogroups 1, 2a, 2b, 2c, and 3 (Figure S9) [38]. The most represented groups were 3 and 1, comprising 15 and 11 isolates, respectively, out of 31. Phylogroup 4 was the only one not represented among the Magarach isolates. 

As a result of the analysis of global GVT diversity, five phylogenetic groups were identified (Figure S10). Two more divergent variants clustered at a distance from the others and may be representatives of the sixth and seventh groups. Magarach isolates belonged to three phylogroups.

The CP sequences of GFkV were divided into two clades (Figure S11). The larger clade (red) included most GFkV isolates, including nine Magarach isolates and all previously published Russian isolates. The smaller clade was subdivided into two groups, one consisting mainly of Croatian isolates (blue), while the other included mostly USA isolates and two Magarach isolates (green).

Among the 11 Magarach GVA isolates, three belonged to phylogroup III and eight to phylogroup I (Figure S12) [39].

Worldwide GVF isolates were divided into large and small clades (Figure S13). The small clade (green) was formed by isolates from South Africa. The large clade (blue) included isolates from multiple geographic regions, including all 11 Magarach isolates.

An analysis of GKSV isolates revealed two clades (Figure S14). Two Magarach isolates clustered in the first clade (green) and were close to Japanese and French isolates. Another Russian isolate belonged to the second clade (blue) and grouped with isolates from Turkmenistan and France. Notably, French isolates were identified in vines originating from Iran.

The Magarach GV-Sat isolates demonstrated high genetic diversity and clustered both with previously published Russian isolates and separately (Figure S15).

Phylogenetic analysis of VCV was performed separately for both genomic RNAs (Figure S16). Clustering of isolates in the resulting dendrograms was consistent. One Magarach VCV isolate clustered together with isolates from France and Russia, whereas the other two formed a group with VCV isolates from wild grapevines of the Black Sea region. 

Magarach isolates of GYSVd-1 and HSVd were analyzed together with representative members of the corresponding phylogroups, while all GenBank sequences available at the time of analysis were included for GYSVd-2 and AGVd. As a result, the analyzed GYSVd-1 isolates belonged to types 1, 2 and 3 (Figure S17) [40]. HSVd isolates belonged to the Hop and Plum-Hop/Cit3 groups (Figure S18) [41]. Phylogenetic analysis of global GYSVd-2 diversity revealed four groups (Figure S19). One group consisted of five Russian isolates and one Chinese isolate. The remaining groups included numerous isolates without clear geographic structure. The only Magarach GYSVd-2 isolate grouped together with two Russian isolates from the Krasnodar region and Dagestan. Worldwide AGVd isolates were divided into two large clades (Figure S20). One clade (blue) included mainly Chinese and Indian isolates. The second clade (red) consisted of isolates from other geographic regions, including two Magarach and other Russian isolates.

### 2.3. Discovery of Novel Grapevine Viruses

#### 2.3.1. Novel Umbra-like Virus

De novo assembly of reads from the C1956 library using the Geneious and SPAdes assemblers generated contigs of 3586 bp and 3088 bp, respectively, that showed identical overlapping regions. TblastX analysis against the NCBI reference viral genome database showed similarity between the 3586 bp contig and the carrot mottle mimic umbravirus (NC_001726; E-value = 4.66 × 10^−118^, identical sites = 16.54%). BlastN analysis against the core nucleotide database showed that the contig was most closely related to the recently described grapevine umbra-like virus 4 (GULV-4; isolate OR947507). A total of 200 reads from the C1956 library were mapped to the identified contig with an average coverage depth of 8.0. Pairwise alignment between the contig and OR947507 showed 69.5% nucleotide identity.

Since the genome length of GULV-4 is 3563 nt, the identified contig was considered to represent a nearly complete genome sequence of a putative novel virus tentatively named grapevine umbra-like virus 5 (GULV-5). RT-PCR using specific primers (Table S5) confirmed the presence of GULV-5 in seven grapevine samples (Table S2).

**Figure 2:**
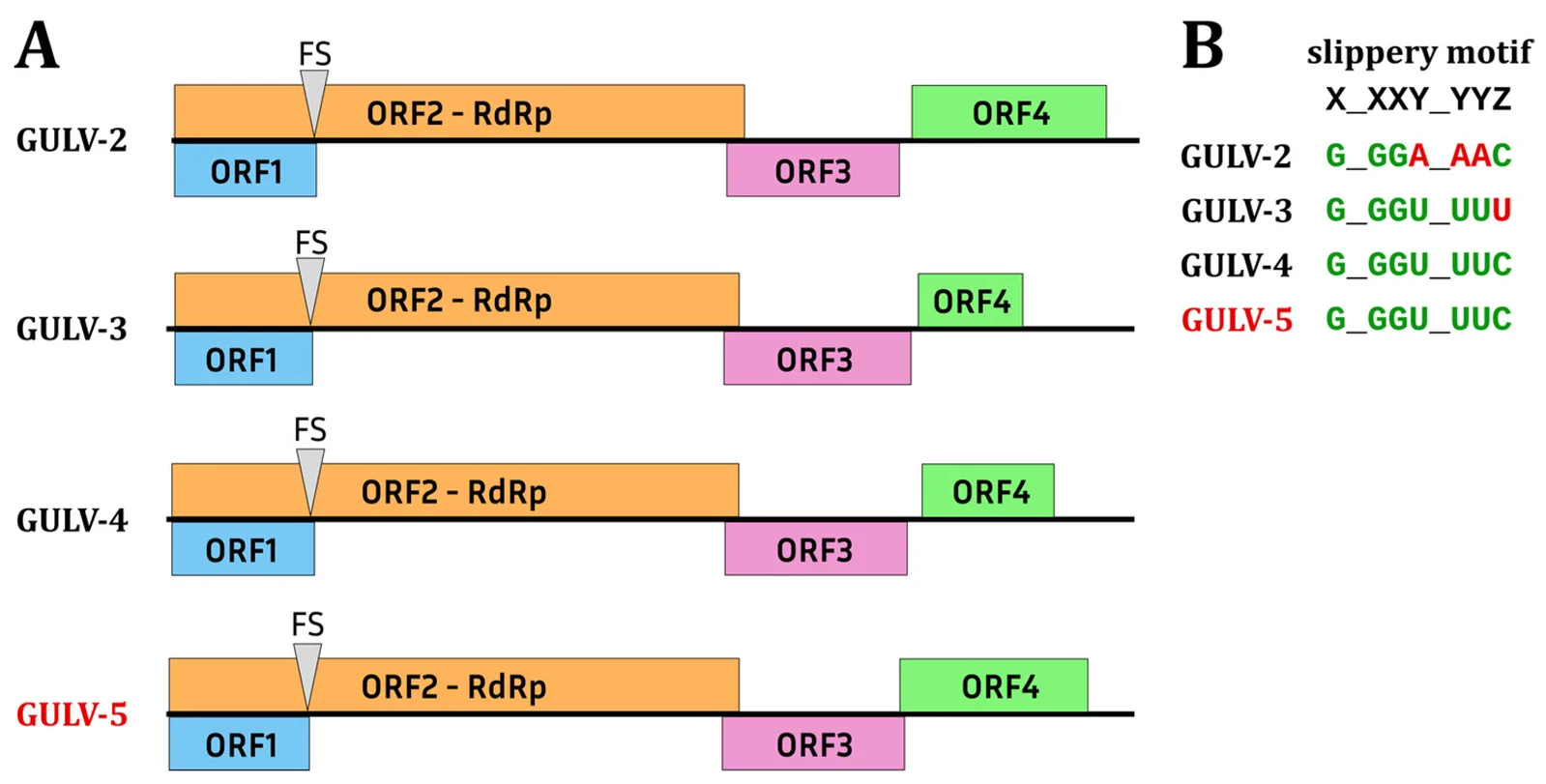
(**A**) Putative genome organization of GULVs. GULV-5 discovered in this study is highlighted in red. ORF1 and ORF2 encode RdRp via the -1 ribosomal frameshift event (FS); ORF3 and ORF4 encode proteins with unknown function. (**B**) Slippery heptamer motif at the end of ORF1 of GULVs that leads to frameshifting.

ORF prediction using ORFfinder identified four ORFs in the GULV-5 genome (Figure 2A). ORF1 encodes a 176 aa protein containing the conserved *Tombusvirus* p33 domain (IPR013707, according to InterPro), which is associated with the replicase of viruses of the family *Tombusviridae*. A heptanucleotide slippery sequence motif (XXXYYYZ) was identified at the 3′ end of ORF1 (Figure 2B), suggesting the presence of programmed −1 ribosomal frameshifting [42]. As a result of the predicted frameshift event, translation extends into ORF2, producing a 705 aa protein containing the RNA-dependent RNA polymerase (RdRp) domain of the (+) RNA viruses (IPR007094). ORF3 partially overlaps with adjacent ORFs, sharing 64 nt with the 3′ end of ORF2 and 19 nt with the 5′ end of ORF4. ORF3 and ORF4 encode proteins of 225 aa and 232 aa, respectively, for which no conserved domains were identified.

**Figure 3:**
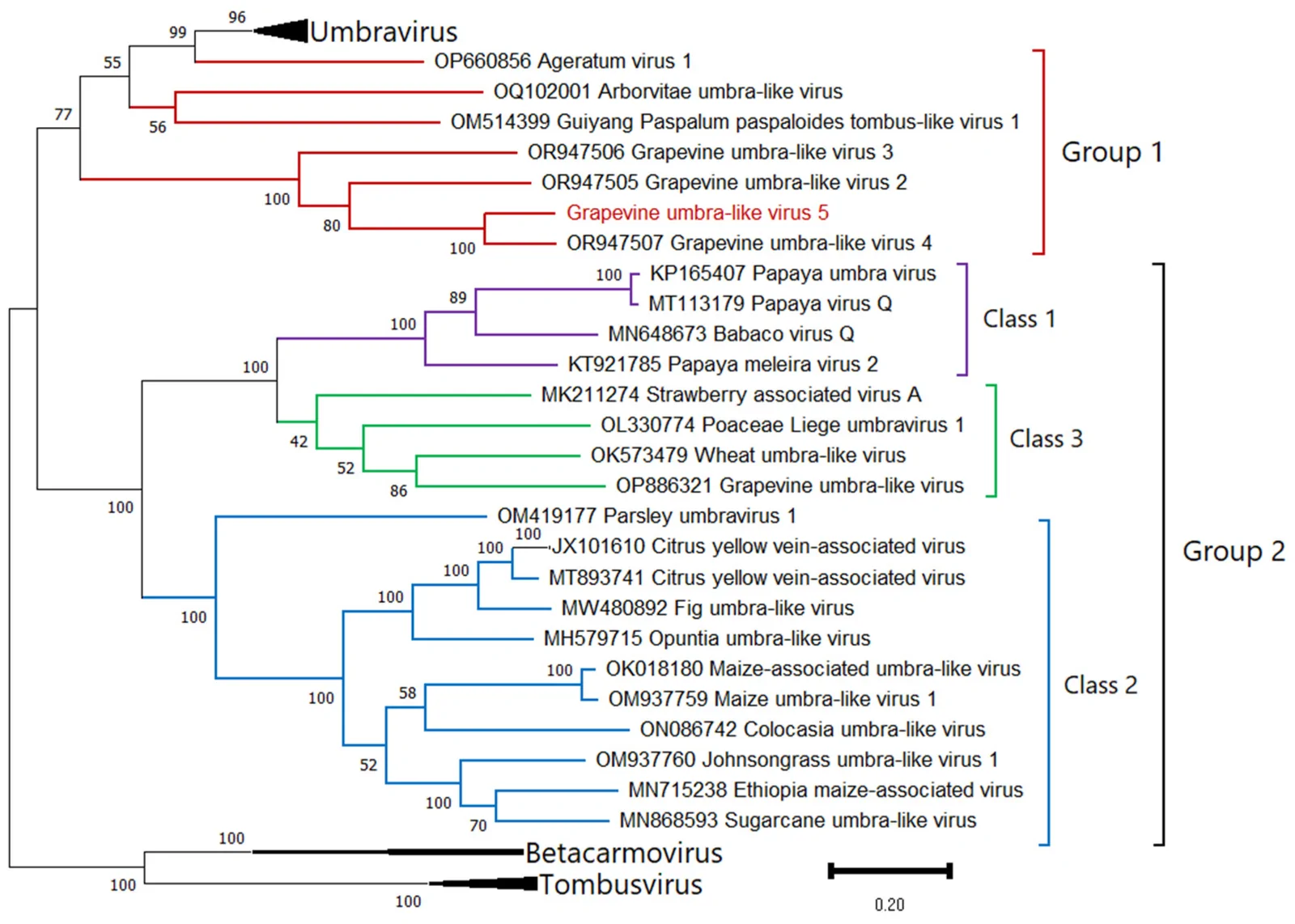
Phylogenetic analysis based on nucleotide sequences of RdRp from umbraviruses and umbra-like viruses. Betacarmoviruses and tombusviruses were used as an outgroup. GULV-5 discovered in this study is highlighted in red. The tree was constructed in MEGA11 using the maximum-likelihood method and the GTR model with 1000 bootstrap replicates.

Phylogenetic analysis of GULV-5 was performed using RdRp nucleotide sequences of representative members of the family *Tombusviridae*, including tombusviruses, betacarmoviruses, umbraviruses, and umbra-like viruses. The resulting phylogenetic tree demonstrated that GULV-2, GULV-3, GULV-4, and GULV-5 form a distinct clade within the umbra-like viruses (Figure 3).

One of the species demarcation criteria established for the ICTV-approved genus *Umbravirus* is a nucleotide sequence identity of less than 70% [43]. The nearly complete genome of GULV-5 shared 69.5% nucleotide identity with its closest relative, GULV-4, and also differed in the organization of ORF3 and ORF4. These findings suggest that GULV-5 may represent a distinct viral species.

#### 2.3.2. Novel Secovirus

TblastX analysis of contigs from the C1960 library showed similarity to cucurbit mild mosaic virus (NC_038760), peach leaf pitting-associated virus (NC_077801), and prunus virus F (NC_039078), with an E-value of up to 1.42 × 10^−19^ and 36–59% identity. Alignment of overlapping contigs enabled the reconstruction of two sequences of 6148 nt and 5993 nt corresponding to RNA1 and RNA2, respectively. BlastN analysis against the core nucleotide database revealed the closest similarities to grapevine secovirus (GSV; OR947508–OR947509) and grapevine fabavirus 2 (GFabV2; BK065046–BK065047). Both of these viruses have bipartite (+) RNA genomes and belong to the family *Secoviridae*. Pairwise alignment showed that the 6148 nt sequence shared 79.0% and 86.4% nucleotide identity with RNA1 of GSV and GFabV2, respectively. The 5993 nt sequence shared 71.1% and 71.2% nucleotide identity with RNA2 of GSV and GFabV2, respectively. These results suggest that the identified sequences represent a divergent isolate related to GSV/GFabV2, hereafter designated GSV-1960.

As a result of PCR validation, both RNAs of this isolate were detected in four samples.

TblastX analysis of contigs assembled from the C1963 library identified two contigs of 5968 nt and 2926 nt showing similarity to members of the family *Secoviridae*: cowpea severe mosaic virus (NC_003545) and peach leaf pitting-associated virus (NC_077800), with an E-value of up to 5.99 × 10^−26^ and an identity of 10.41–11.28%. BlastN searches against the core nucleotide database yielded no matches. Subsequent BlastX analysis demonstrated that the 5968 nt and 2926 nt contigs were most closely related to RNA1 and RNA2, respectively, of members of the genus *Fabavirus* (family *Secoviridae*). This suggested that the identified contigs corresponded to the genomic segments of a novel virus tentatively named grapevine Magarach secovirus (GMSV).

Mapping of reads from all libraries to the RNA1 and RNA2 sequences enabled the detection of GMSV in three libraries, which were confirmed by RT-PCR (Table S2).

**Figure 4:**
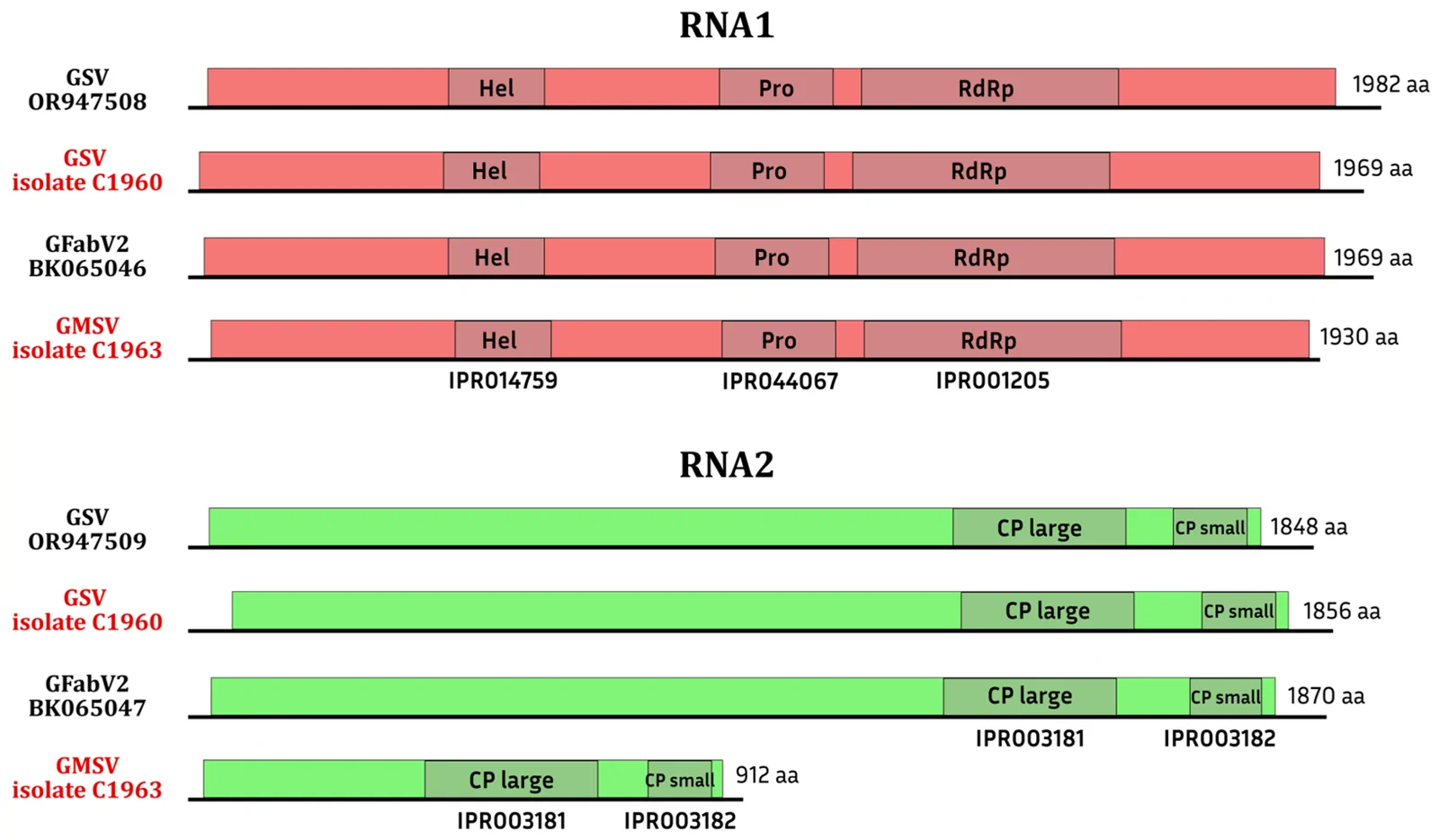
Putative genome organization of GMSV and related secoviruses. Isolates discovered in this study are highlighted in red. Each ORF encodes a polyprotein. The dark rectangles indicate conserved domains predicted by InterPro.

The new secovirus isolates GMSV and GSV-1960 discovered in this study contained a single ORF on both RNA1 and RNA2, encoding polyproteins 1 and 2, respectively (Figure 4). Polyprotein 1 is 1930 aa in length in GMSV and 1969 aa in GSV-1960 and contains three conserved domains: helicase (IPR014759), proteinase (IPR044067), and polymerase (IPR001205). Polyprotein 2 is 912 aa in GMSV and 1856 aa in GSV-C1960 and contains conserved domains of capsid proteins that are typical of comoviruses and fabaviruses (IPR003181 and IPR003182).

**Figure 5:**
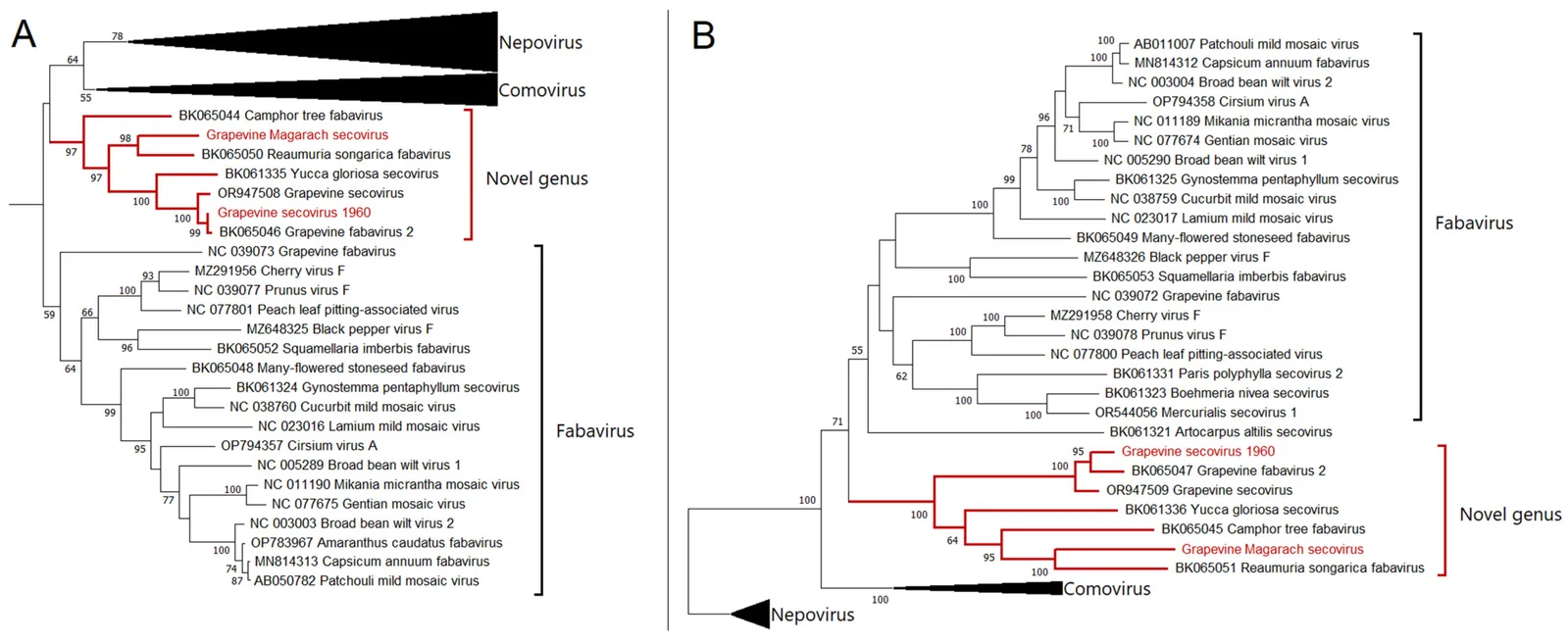
Phylogenetic analysis based on amino acid sequences of (**A**) the Pro-Pol region or (**B**) polyprotein 2 of members of the genera *Nepovirus*, *Fabavirus*, *Comovirus*, and unclassified *Secoviridae*. Isolates discovered in this study are highlighted in red. The trees were constructed in MEGA11 using the maximum-likelihood method and (**A**) the LG or (**B**) the WAG models with 1000 bootstrap replicates.

Phylogenetic analysis was performed based on the amino acid sequences of the Pro-Pol region of RNA1 (Figure 5A) and the polyprotein of RNA2 (Figure 5B) from representatives of the genera *Nepovirus*, *Fabavirus*, *Comovirus*, and unclassified *Secoviridae*. In both dendrograms, GMSV, GSV, GFabV2, yucca gloriosa secovirus (YgSV), camphor tree fabavirus (CtFabV), and reaumuria songarica fabavirus (RsFabV) formed a separate clade located between the comovirus and fabavirus clades. The virus most closely related to GMSV was RsFabV, which was previously discovered using transcriptome analysis of the plant *Reaumuria soongarica* [44]. GFabV2 and the two GSV isolates clustered together in both phylogenetic analyses.

Amino acid sequences of the Pro-Pol region and CP (large and small domains) from phylogenetically related viruses and representative members of the genus *Fabavirus* were used for SDT analysis (Tables S6 and S7). The Pro-Pol and CP amino acid sequences of GMSV shared 36.2–66.0% and 8.1–32.0% identity, respectively, with those of the analyzed viruses. In contrast, the conserved Pro-Pol regions of the two GSV isolates and GFabV2 shared 92.5–97.9% amino acid identity. The CP sequences of these three isolates shared 87.3–91.8% amino acid identity, whereas CP identity among other viruses within the same clade ranged from 9.3% to 32.0%.

The currently accepted species demarcation criteria for members of the family *Secoviridae* include less than 80% amino acid identity within the conserved Pro-Pol region and less than 75% amino acid identity within the CP region [45]. The Pro-Pol and CP sequences of GMSV were substantially more divergent from those of related viruses. These findings support the classification of GMSV as a putative novel species within the family *Secoviridae*.

## 3. Discussion

In this study, two novel grapevine viruses were identified and characterized. The first, GULV-5, represents an additional umbra-like virus along with those previously discovered on grapevines from Russian ampelographic collections [22,23,24]. All five currently known GULVs belong to the recently classified group of umbra-like viruses (ULVs), previously called umbravirus-like associated RNAs (ulaRNAs) [46,47]. According to the classification proposed by the authors, GULV-2, GULV-3, GULV-4, and GULV-5 belong to Group 1 of ULVs (Figure 3). This group is phylogenetically closer to umbraviruses than other ULVs.

ULVs are similar to umbraviruses in the genomic organization of ORF1 and ORF2 but do not contain ORFs encoding cell-to-cell and long-distance MPs. In both umbraviruses and ULVs, the replicase is translated from ORF1 and ORF2 via the −1 ribosomal frameshift event [46]. The slippery heptamer motif required for frameshifting was identified at the 3′ end of ORF1 in all GULVs belonging to Group 1, including GULV-5 (Figure 2B). Therefore, we assume that replicase translation in GULV-5 occurs via the same mechanism. The genomic organization of GULV-5 is similar to that of other GULVs. The main difference from them is the overlap of ORF3 and ORF4 by 19 nt. Recent structural and phylogenetic analyses predicted a full jelly roll fold for the GULV-5 ORF4-encoded protein and suggested that it may represent a capsid-like protein [48]. However, this putative function remains experimentally unconfirmed.

Umbraviruses require the presence of a helper virus from the family *Solemoviridae* for productive infection [49]. However, no members of this family were detected in grapevines from the Magarach ampelographic collection. Similar observations were previously reported during the discovery of other GULVs and most ULVs [50,51]. Recently, Ying et al. provided evidence that ULVs may use phloem proteins for movement and therefore do not need helper viruses [52]. GULV-5 may employ a similar mechanism for movement within the host plant.

Recent work demonstrated that GULV-4 is capable of systemic infection of *Nicotiana benthamiana* without functional ORF3 or ORF4 [48]. Mutant variants carrying premature termination codons in ORF3 or ORF4 were still detected in systemic leaves, although only the infiltrated leaves developed necrotic symptoms, indicating that these ORFs are not required for systemic movement. Considering the close phylogenetic relationship between GULV-4 and GULV-5, it is possible that GULV-5 may also have pathogenic potential. Further studies are required to determine its ability to infect grapevine systemically and to evaluate its possible association with disease symptoms.

Species demarcation criteria for ULVs have not yet been established, but according to the currently accepted criteria for the genus *Umbravirus* (nt identity < 70%) [43], together with differences in genome organization relative to the closest described virus, GULV-5 likely represents a distinct species within Group 1 of ULVs.

In addition to GULV-5, this study identified and characterized GMSV, a novel bipartite (+) RNA virus related to members of the family *Secoviridae*. Many secoviruses are considered economically important plant pathogens; for example, GFLV is one of the most damaging viral pathogens of grapevine [53]. Due to the presence of mixed infection, it was not possible to associate GMSV with specific symptoms.

Another secovirus identified in this study, GSV-1960, was closely related to a previously characterized GSV [24] and GFabV2 identified through grapevine transcriptome analysis [44]. All three isolates have a similar genome organization (Figure 4), with only minor differences in ORF lengths. Phylogenetic (Figure 5) and SDT (Tables S6 and S7) analyses demonstrated high sequence similarity among these isolates and supported their classification within the same viral species, GSV.

Phylogenetic analysis of the amino acid sequences of the conserved Pro-Pol region showed that GSV, YgSV, GFabV2, CtFabV, GMSV, and RsFabV form a distinct clade separate from the related genera *Comovirus* and *Fabavirus*. Interestingly, isolates within this clade differed in genome length. In GSV, YgSV, GFabV2, and CtFabV, RNA2 ranged from 5477 to 6739 nt in length, which is approximately 2500 nt longer than the RNA2 segments of members of the genus *Fabavirus* [45]. In contrast, GMSV and RsFabV possessed RNA2 segments of a size more typical of fabaviruses. Taken together, these findings suggest that this group of viruses may represent a distinct genus within the family *Secoviridae*.

**Figure 6:**
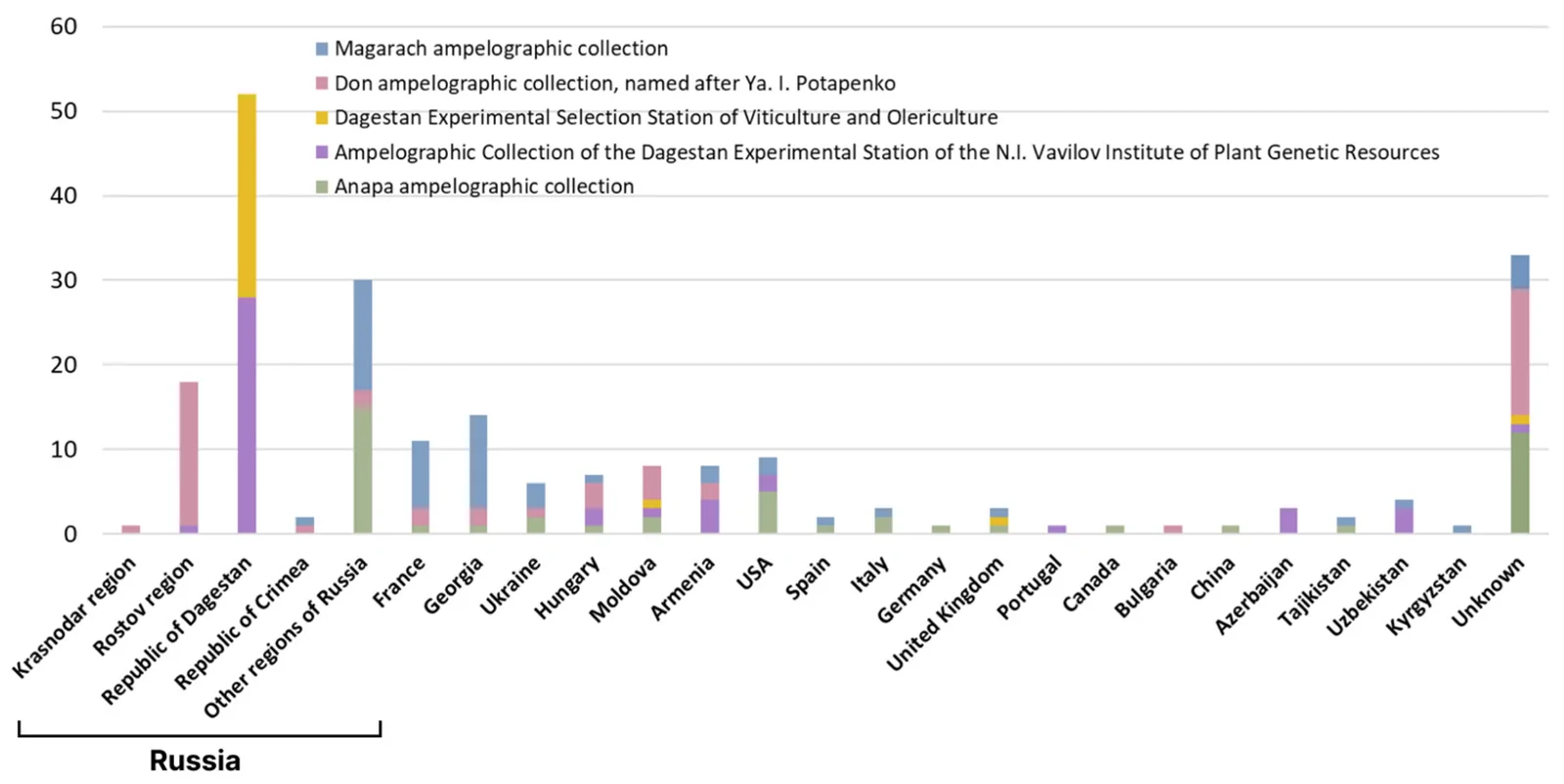
Country of origin of the grapevine varieties used in the research on ampelographic collections.

This study completes a series of virome analyses of the five main ampelographic collections in Russia [22,23,24]. In total, 222 grapevines were analysed, including varieties originating from the main Russian breeding centers, promising breeding lines, widely cultivated varieties, and unique indigenous varieties growing in the Krasnodar region, the Rostov region, the Republic of Dagestan, and other regions (Figure 6).

**Figure 7:**
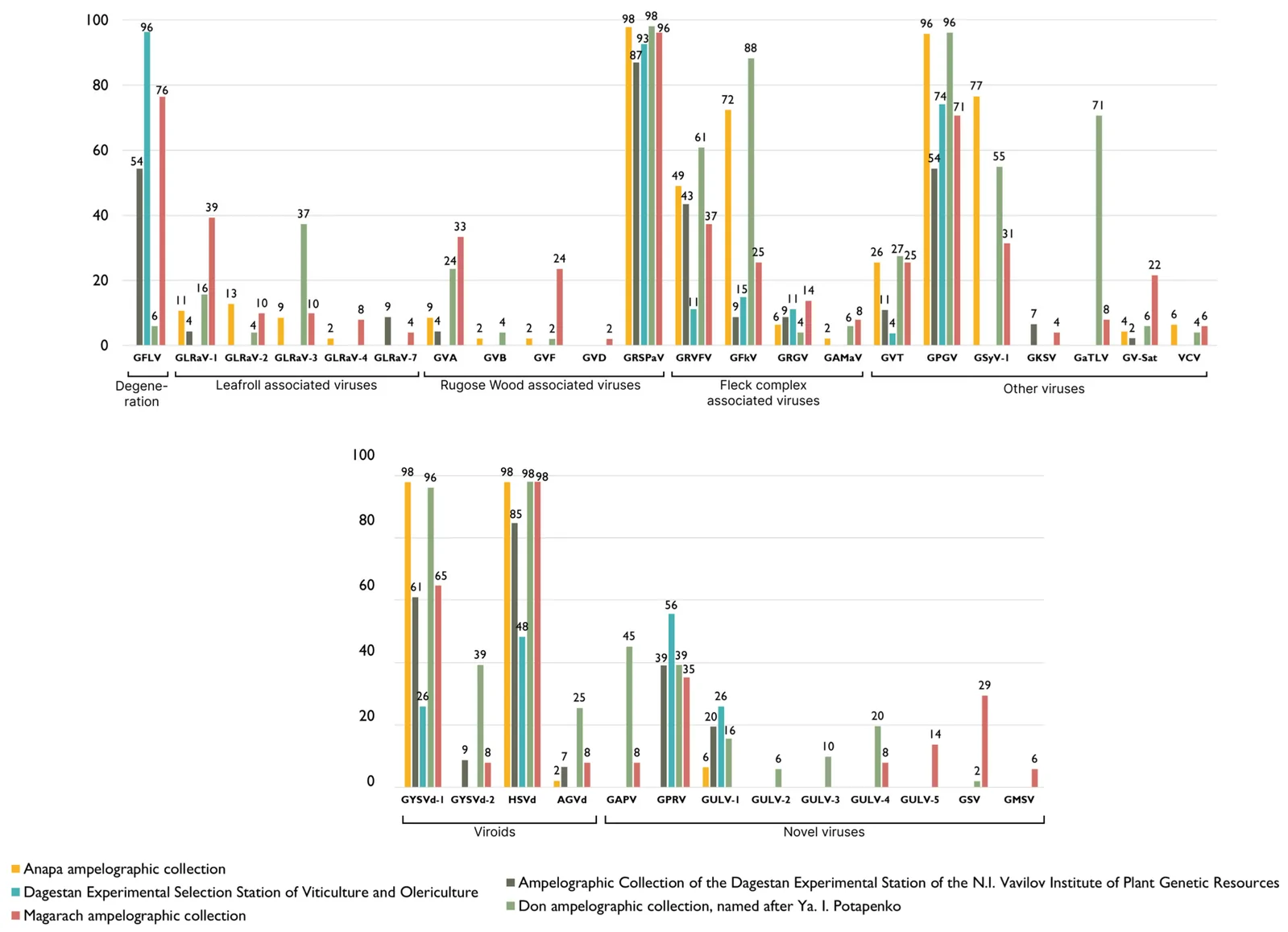
Distribution of viruses and viroids in grapevines from five ampelographic collections in Russia (percentage of the total number of samples collected in each collection).

Altogether, 31 virus species and four viroid species were identified (Figure 7). Mixed infections involving one to sixteen viruses and viroids were detected in the analyzed grapevines. The highest number of virus and viroid detections (512) was recorded in the Don ampelographic collection, whereas the lowest number (124) was observed in the Dagestan Experimental Selection Station of Viticulture and Olericulture (Table S3).

**Figure 8:**
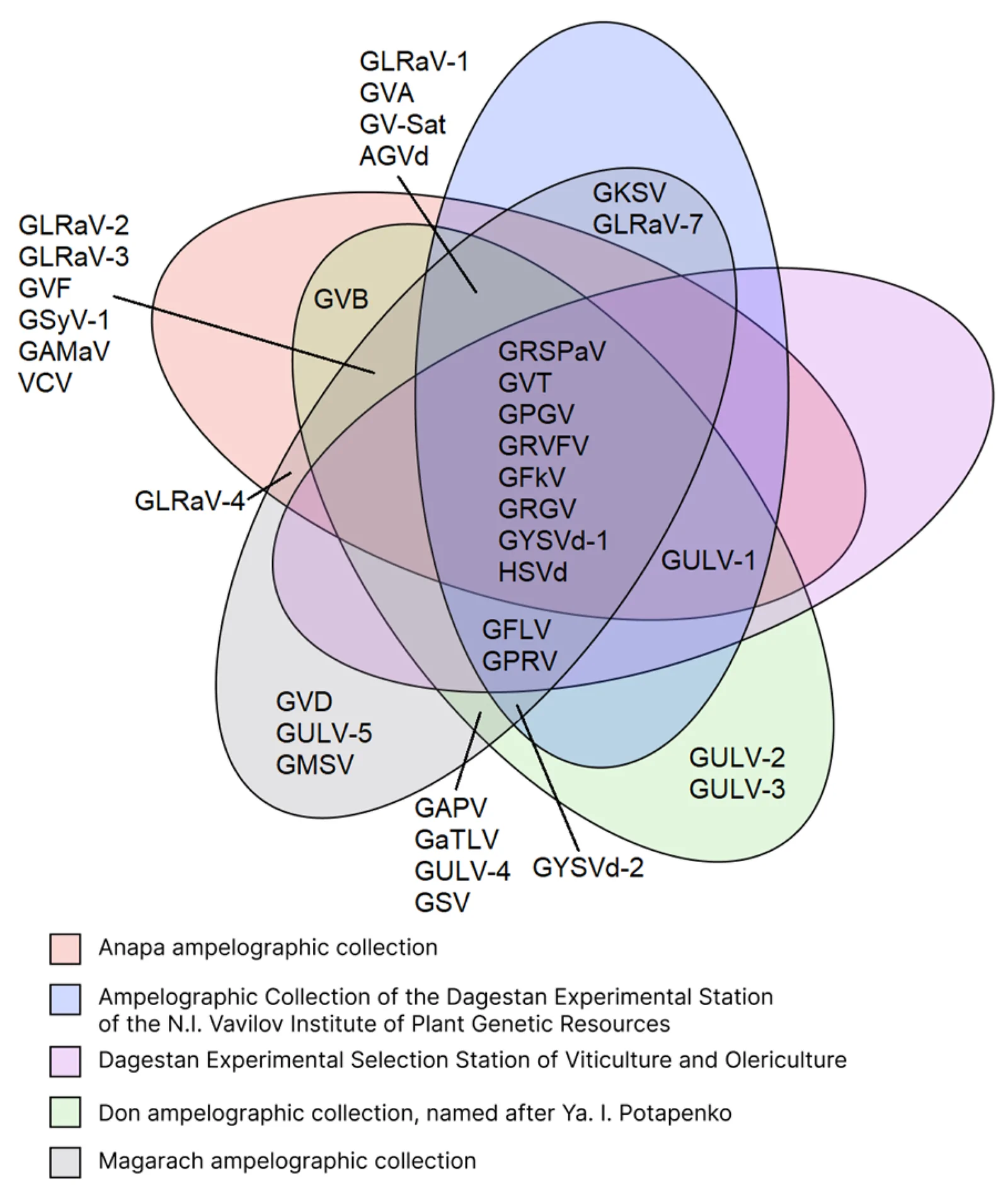
Number of grapevine viruses and viroids detected in five ampelographic collections in Russia.

Although the species composition and prevalence differed among collections, six viruses and two viroids were detected in all analyzed collections (Figure 8). The most prevalent viruses were GRSPaV, detected in 95% of grapevines, and GPGV, detected in 79% (Table S3). Other viruses shared among all collections included GVT (20%), GRVFV (43%), GFkV (45%), and GRGV (9%). Among viroids, HSVd (86%) and GYSVd-1 (73%) were the most widespread.

Among economically important viruses, GFLV is considered the oldest viral pathogen of grapevine and is associated with severe symptoms, including malformations, yellow mosaic, shortened internodes, double nodes, and zig-zag growth [53]. GFLV is transmitted by the nematode *Xiphinema index*, which can retain virus particles in the soil for at least four years [54]. In the analyzed ampelographic collections, GFLV was detected more frequently than other economically important viruses and was identified in 42% of the analyzed grapevines. However, GFLV was not detected in the samples from the Anapa collection.

Another economically important disease detected in the collections was grapevine leafroll disease (GLD). Among the associated viruses, GLRaV-1 (16%) and GLRaV-3 (13%) were the most prevalent. GLRaV-3 was detected in all collections except those from Dagestan. GLRaV-2 was identified in 6% of grapevines but was also absent from the Dagestan collections.

In this series of studies, we identified ten grapevine viruses that had not previously been reported in Russia and discovered nine novel viral species, the most prevalent of which was GPRV, detected in 32% of analyzed grapevines. Five of the discovered viruses belonged to the group of umbra-like viruses, which had not previously been described in grapevines. One of them, GULV-1, was detected in 12% of the analyzed grapevines. The biology, pathogenicity, epidemiology, distribution in commercial vineyards, and mechanisms of gene expression of these novel viruses remain to be investigated in future studies.

**Figure 9:**
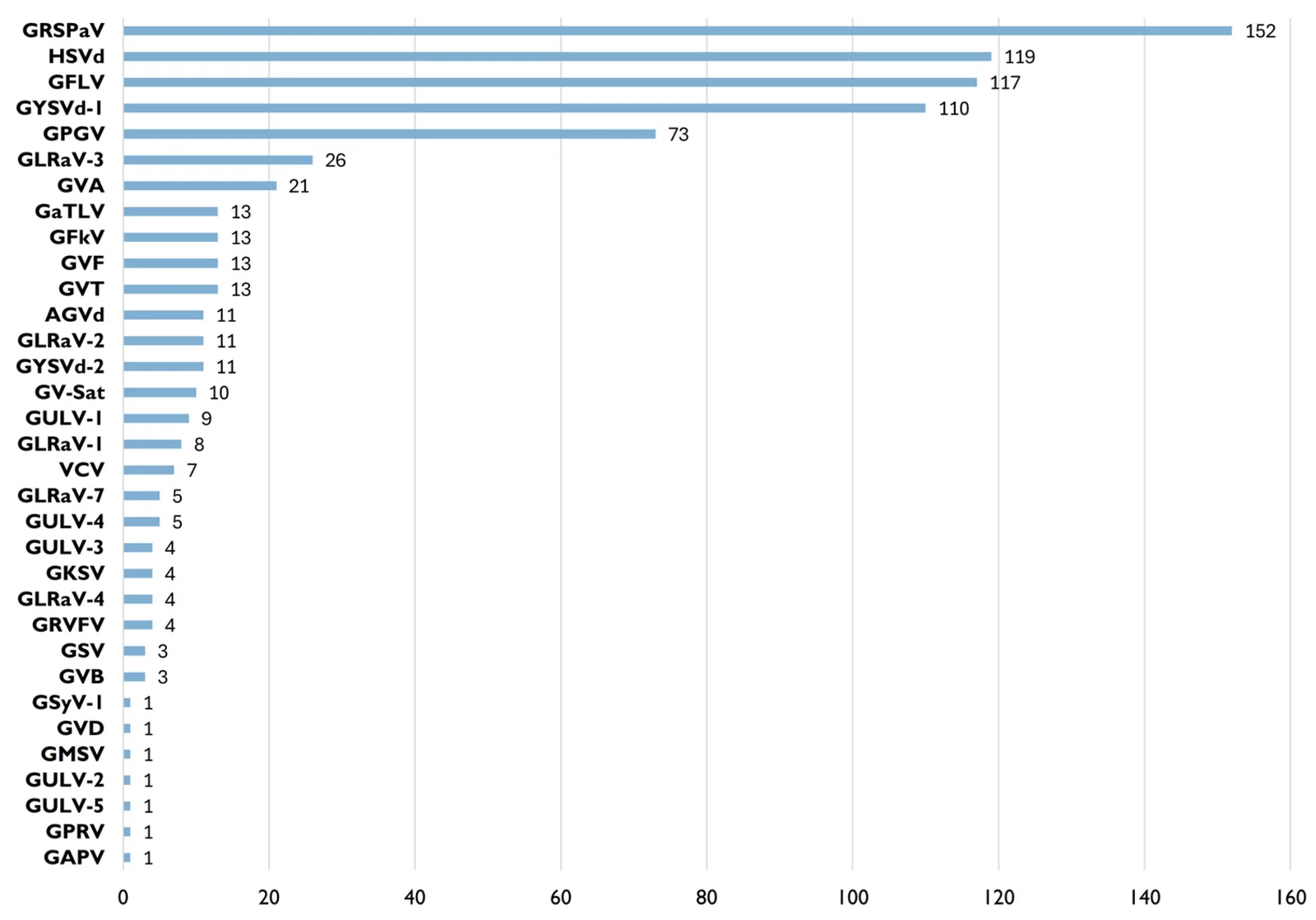
Number of assembled genomes of grapevine viruses and viroids in five ampelographic collections in Russia.

The use of metagenomic analysis enabled not only the assessment of virus species composition and distribution but also the assembly of 776 complete or nearly complete genomes representing twenty-nine viruses and four viroids (Figure 9). These data substantially expand the genomic resources available for studies of grapevine virus epidemiology and population genetics. In particular, phylogenetic analysis revealed the presence in Russia of GRSPaV isolates belonging to all six currently distinguished phylogroups [38]. Grapevine viroids also demonstrated high genetic diversity: isolates of all four described molecular types of GYSVd-1 [40] and three HSVd groups (Hop, Plum-Hop/Cit3, and Citrus) [41] were identified.

For a number of viruses, such as GVA and GLRaV-2, determining the phylogroup is important because different molecular groups may differ in pathogenicity. For example, GLRaV-2 isolates are divided into six phylogroups [29,30]. Among them, the BD group is generally considered asymptomatic [55], whereas the PN group is associated with leafroll symptoms [56]. Four GLRaV-2 phylogroups (H4, PV20, BD, and PN) were identified in the analyzed Russian ampelographic collections. Similarly, GVA molecular variants are classified into three phylogroups, one of which (group II) has been associated with Shiraz disease [39]. In the Russian grapevine germplasm, only GVA phylogroups I and III have been identified. These molecular variants are typically associated with asymptomatic infections [57,58].

The importance of studying the virus genome for the identification of potentially virulent variants can be illustrated by GPGV. Although GPGV is frequently detected in asymptomatic grapevines, under certain conditions it may be associated with grapevine leaf mottling and deformation (GLMD) [59]. The symptom severity is correlated with the presence of an early stop codon at the end of the reading frame encoding MP [35,60,61]. Comparative analysis of this region in Magarach GPGV isolates using representative virulent and latent strains [35,36,37] demonstrated the absence of the early stop codon in the analyzed isolates (Figure S8). A similar observation was previously reported for other Russian GPGV isolates [62], indicating the absence of virulent GPGV strains in Russia.

Metagenomic analysis also enabled detailed characterization of the genetic diversity of Russian grapevine viruses in the context of global populations. Phylogenetic analysis of Russian GLRaV-3 isolates identified representatives of two out of the nine currently described phylogroups [31,32,33]. Notably, the Magarach collection included an isolate clustering with the divergent GLRaV-3m variant, which had previously been considered a separate species, GLRaV-12 [63]. Since only a limited portion of the known global diversity of GLRaV-3 appears to be represented in Russia, continued phytosanitary control of imported planting material remains important.

Although GLRaV-1 and GLRaV-3 are considered the principal viral agents associated with leafroll disease [64], other GLRaVs may also contribute to disease development. In this study, the GLRaV-4 strain Ob was detected in Russia for the first time. This strain has previously been reported to induce mild leafroll symptoms in the absence of other GLRaVs [34].

Phylogenetic analysis of GFLV, GPGV, and GVT genomes detected in the Russian grapevine germplasm provided insights into the phylogeography of these viruses.

Phylogenetic analysis of GFLV revealed pronounced geographic structuring of isolates (Figures S1 and S2). Analysis of RNA1 sequences identified a distinct clade composed predominantly of Russian isolates, most of which were identified in grapevines from the Dagestan ampelographic collections. Phylogenetic analysis of RNA2 sequences demonstrated relationships between isolates from this clade and isolates from Japan, China, and Iran. These findings suggest separation of the global GFLV population into Asian- and European-associated genetic groups. This observation is consistent with the fact that most varieties in the Dagestan collections are of local origin. In addition, many grapevine accessions maintained in the Magarach collection historically originated from Asian germplasm.

GPGV is believed to have been introduced to Europe from Asia [65], probably from wild Far Eastern *V. coignetiae* [66]. One isolate identified in Japanese *V. coignetiae* (LC601811) was included in our phylogenetic analysis and, together with several Chinese isolates, formed a cluster distinct from Russian and European isolates (Figure S7). These findings suggest that GPGV currently circulating in Russian vineyards was likely introduced through infected European planting material. At the same time, GPGV isolates identified in wild *V. vinifera* ssp. *sylvestris* from the Black Sea region clustered together with cultivated Russian isolates, which may indicate recent transmission of GPGV from vineyards into forest ecosystems [67].

Phylogenetic analysis of GVT genetic variants expanded current knowledge of the global diversity of this virus and prompted a reassessment of previously proposed phylogroup assignments [68]. Our results suggest that the genetic diversity of GVT is greater than previously described. Some isolates formed distinct phylogenetic groups, whereas some previously proposed groups appeared insufficiently differentiated and may warrant consolidation (Figure S10). Continued expansion and increasing accessibility of metagenomic sequencing are expected to further improve our understanding of the phylogeography of GVT and other grapevine viruses.

## 4. Materials and Methods

### 4.1. Sampling and Preparation of Total RNA Libraries

The Magarach ampelographic collection was surveyed for symptom observation and sampling in 2020. A total of 51 grapevine samples exhibiting symptoms associated with viral infection were selected for analysis (Table S8). Total RNA was extracted from 1 g of mixed vine and leaf tissue using the CTAB-LiCl method described by Morante-Carriel et al. [69]. RNA quality was assessed using a BioSpectrometer spectrophotometer (Eppendorf, Hamburg, Germany) and electrophoresis in 1.2% agarose gel. Purified nucleic acid samples were treated with DNase I (Thermo Fisher Scientific, Waltham, MA, USA) and the RiboMinus Plant Kit (Thermo Fisher Scientific, Waltham, MA, USA). RNA concentration was measured using a Qubit 4.0 fluorimeter (Invitrogen, Waltham, MA, USA) and the Qubit RNA BR Assay Kit (Thermo Fisher Scientific, Waltham, MA, USA). cDNA libraries were prepared using the QIAseq Stranded RNA Library Kit (Qiagen, Hilden, Germany). Library quality was assessed using a Bioanalyzer 2100 (Agilent Technologies, Santa Clara, CA, USA). DNA concentration was measured using the HS DNA Assay Kit (Thermo Fisher Scientific, Waltham, MA, USA). Sequencing was performed on a NovaSeq 6000 System (Illumina, San Diego, CA, USA), generating 150 bp paired-end reads. Raw FASTQ sequencing data were deposited in the Sequence Read Archive (SRA) under accession number PRJNA1191642.

### 4.2. HTS Data Analysis and Assembly of Viral Genomes

Bioinformatics analysis of sequencing data was performed using Geneious Prime v. 2023.2.1 (Biomatters, Auckland, New Zealand). Low-quality reads (minQ = 30), short reads (<10 bp), and adapter sequences were removed using the BBDuk tool. The filtered reads were merged and deduplicated at kmer = 30. Preprocessed reads were assembled into contigs using RNA SPAdes v. 3.15.5 with default settings and the Geneious assembler with medium-low sensitivity. Contigs were annotated using the TblastX algorithm against the NCBI Viral Reference Genome Database (release date: 9 March 2024). The E-value threshold for contig annotation was set to 1 × 10^−40^.

In parallel with de novo assembly, preprocessed reads were mapped to reference genomes of viruses and viroids (accessed on 27 March 2026) or to genomes of the closest isolates with medium-low sensitivity, except for cases listed in Table S9. Viral genome sequences were assembled using either whole-genome contigs, when available, or consensus sequences after mapping. Assembled genome sequences were deposited in GenBank under the accession numbers listed in Table S10.

To minimize false-positive virus identification in libraries containing more than 1 million preprocessed reads, detection thresholds of at least 10 mapped reads and 15% reference genome coverage were applied to viruses with high read abundance, including GRSPaV, GVT, GPGV, GLRaVs, GFkV, GVA, GVD, GVF, GKSV, and GFLV. Samples were considered virus-positive only when virus detection was confirmed by both bioinformatic analysis and PCR.

### 4.3. Discovery and Analysis of Novel Grapevine Viruses

Contigs showing similarity to unexpected plant viruses in the TblastX analysis were further verified using BlastN against the core nucleotide database and BlastX against the nr protein database in GenBank. Sequences sharing less than 80% nucleotide identity with known viral sequences were selected for further analysis. Contig orientation was determined by alignment with the genomes of the closest viruses using the Clustal Omega v. 1.2.2 algorithm. To verify assembly accuracy, preprocessed reads from the corresponding libraries were mapped back to the assembled contigs.

Genome annotation of putative novel viruses was performed using the ORFfinder tool. Conserved protein domains were predicted using InterPro [70].

Pairwise sequence comparisons between the novel viruses identified in this study and related viral species available in GenBank were performed using the ClustalW algorithm in the Sequence Demarcation Tool (SDT) [71].

### 4.4. Validation of Grapevine Viruses and Viroids

RT-PCR and RT-qPCR assays were performed to validate the viruses and viroids detected by bioinformatic analysis. Reverse transcription was carried out using random hexamer primers and MMLV reverse transcriptase (Evrogen, Moscow, Russia) according to the manufacturer’s instructions. Successful cDNA synthesis was verified by amplification of a fragment of the 18S rRNA gene, which was used as an internal control. PCR was performed with Encyclo polymerase (Evrogen, Moscow, Russia) on a T100 Thermal Cycler (Bio-Rad, Berkeley, CA, USA). The PCR mix contained 7.7 µL of deionized water, 1.5 µL of each primer (10 mM), 1.5 µL of 10X Encyclo buffer, 1.5 µL of dNTP mix, 0.3 µL of 50X Encyclo polymerase, and 1 µL of cDNA. PCR products were analyzed by electrophoresis in 1% agarose gel. Primers used for virus and viroid validation are listed in Table S5. Primers for detection of novel viruses were designed manually and evaluated for potential secondary structures using Beacon Designer Free (Premier Biosoft International, San Francisco, CA, USA).

Detection of GYSVd-1 and HSVd was performed using TaqMan RT-qPCR with the BioMaster HS-qPCR kit (Biolabmix, Novosibirsk, Russia). Reactions were conducted in three technical replicates on a DTprime real-time PCR system (DNA-technology, Moscow, Russia). The resulting data were analyzed using the instrument software.

### 4.5. Phylogenetic Analysis of Viruses and Viroids

Nucleotide sequences of grapevine viruses and viroids assembled in this study together with sequences downloaded from GenBank were used for phylogenetic analysis. Multiple sequence alignment was performed using the Clustal Omega v. 1.2.2 algorithm. The best-fit nucleotide substitution models and maximum likelihood phylogenetic trees were generated in MEGA11 [72]. Bootstrap support included 1000 replicates. Trees were rooted using sequences of the closest related species. Parameters used for each phylogenetic tree are provided in Table S4.

## 5. Conclusions

Ampelographic collections represent reservoirs of both known and previously undescribed grapevine viruses and viroids, some of which may spread to commercial vineyards. The present study demonstrated a high level of virome diversity in Russian grapevine germplasm, including economically important viruses, genetically diverse molecular variants, and two putative novel viral species.

The obtained results highlight the importance of systematic phytosanitary monitoring of grapevine genetic collections and the implementation of sanitary control measures aimed at preserving grapevine germplasm health. Comprehensive virome analysis based on high-throughput sequencing represents an effective tool for virus diagnostics, characterization of viral diversity, and detection of latent or previously unknown pathogens. The capabilities of HTS-based diagnostics should be used for preliminary diagnostics and, if necessary, virus elimination from accessions before adding them to the collection.

## Figures and Tables

**Table 1:** Taxonomic diversity of viruses and viroids identified in grapevines from the Magarach ampelographic collection.

Family	Genus	Species	Common Name	Acronym	No. of Positive Samples
*Betaflexiviridae*	*Foveavirus*	*F. rupestris*	Grapevine rupestris stem pitting-associated virus	GRSPaV	49
		*F. tafvitis*	Grapevine virus T	GVT	13
	*Trichovirus*	*T. pinovitis*	Grapevine Pinot gris virus	GPGV	36
	*Vitivirus*	*V. deltavitis*	Grapevine virus D	GVD	1
		*V. phivitis*	Grapevine virus F	GVF	12
		*V. alphavitis*	Grapevine virus A	GVA	17
	n/a	n/a	Grapevine Kizil Sapak virus	GKSV	2
*Caulimoviridae*	n/a	n/a	Grapevine pararetrovirus	GPRV	18
*Closteroviridae*	*Ampelovirus*	*A. univitis*	Grapevine leafroll-associated virus 1	GLRaV-1	20
		*A. trivitis*	Grapevine leafroll-associated virus 3	GLRaV-3	5
		*A. tetravitis*	Grapevine leafroll-associated virus 4	GLRaV-4	4
	*Closterovirus*	*C. vitis*	Grapevine leafroll-associated virus 2	GLRaV-2	5
	*Velarivirus*	*V. septemvitis*	Grapevine leafroll-associated virus 7	GLRaV-7	2
*Partitiviridae*	*Alphapartitivirus*	n/a	Grapevine alphapartitivirus	GAPV	4
	*Deltapartitivirus*	n/a	Vitis cryptic virus	VCV	3
*Secoviridae*	*Fabavirus*	*F. betavitis*	Grapevine secovirus	GSV	15
	*Nepovirus*	*N. foliumflabelli*	Grapevine fanleaf virus	GFLV	39
*Tombusviridae*	n/a	n/a	Grapevine umbra-like virus 4	GULV-4	4
*Tymoviridae*	*Maculavirus*	n/a	Grapevine Red Globe virus	GRGV	7
		*M. vitis*	Grapevine fleck virus	GFkV	13
	*Marafivirus*	n/a	Grapevine rupestris vein feathering virus	GRVFV	19
		*M. asteroides*	Grapevine asteroid mosaic-associated virus	GAMaV	4
		*M. syrahense*	Grapevine Syrah virus 1	GSyV-1	16
	n/a	n/a	Grapevine-associated tymo-like virus ^1^	GaTLV	4
n/a	*Virtovirus*	n/a	Grapevine satellite virus	GV-Sat	11
*Pospiviroidae*	*Apscaviroid*	*A. alphaflavivitis*	Grapevine yellow speckle viroid 1	GYSVd-1	33
		*A. betaflavivitis*	Grapevine yellow speckle viroid 2	GYSVd-2	4
		*A. austravitis*	Australian grapevine viroid	AGVd	4
	*Hostuviroid*	*H. impedihumuli*	Hop stunt viroid	HSVd	50

^1^ The host of GaTLV has not been conclusively established and remains under debate.

## Data Availability

The raw sequencing data are available in the NCBI SRA repository under the BioProject accession number PRJNA1191642.

## Supplementary Materials

The following supporting information can be downloaded at: https://www.mdpi.com/article/10.3390/plants15172698/s1, Figure S1: Phylogenetic analysis based on sequences of RNA1 of grapevine fanleaf virus (GFLV); Figure S2: Phylogenetic analysis based on sequences of RNA2 of grapevine fanleaf virus (GFLV); Figure S3: Phylogenetic analysis based on genome sequences of grapevine leafroll-associated virus 2 (GLRaV-2); Figure S4: Phylogenetic analysis based on genome sequences of grapevine leafroll-associated virus 3 (GLRaV-3); Figure S5: Phylogenetic analysis based on genome sequences of grapevine leafroll-associated virus 4 (GLRaV-4); Figure S6: Phylogenetic analysis based on genome sequences of grapevine leafroll-associated virus 7 (GLRaV-7); Figure S7: Phylogenetic analysis based on genome sequences of grapevine Pinot gris virus (GPGV); Figure S8: Phylogenetic analysis based on a 549 bp segment of the MP/CP gene of grapevine Pinot gris virus (GPGV); Figure S9: Phylogenetic analysis based on genome sequences of grapevine rupestris stem pitting-associated virus (GRSPaV); Figure S10: Phylogenetic analysis based on genome sequences of grapevine virus T (GVT); Figure S11: Phylogenetic analysis based on the CP gene of grapevine fleck virus (GFkV); Figure S12: Phylogenetic analysis based on genome sequences of grapevine virus A (GVA); Figure S13: Phylogenetic analysis based on genome sequences of grapevine virus F (GVF); Figure S14: Phylogenetic analysis based on genome sequences of grapevine Kizil Sapak virus (GKSV); Figure S15: Phylogenetic analysis based on genome sequences of grapevine satellite virus (GV-Sat); Figure S16: Phylogenetic analysis based on sequences of RNA1 and RNA2 of Vitis cryptic virus (VCV); Figure S17: Phylogenetic analysis based on genome sequences of grapevine yellow speckle viroid 1 (GYSVd-1); Figure S18: Phylogenetic analysis based on genome sequences of hop stunt viroid (HSVd); Figure S19: Phylogenetic analysis based on genome sequences of grapevine yellow speckle viroid 2 (GYSVd-2); Figure S20: Phylogenetic analysis based on genome sequences of Australian grapevine viroid (AGVd); Table S1: Number of reads and contigs obtained for each library after sequencing by the Illumina NovaSeq 6000 Sequencing System; Table S2: Bioinformatics analysis and its validation; Table S3: Summarised data on the number of identified grapevine viruses and viroids in five ampelographic collections in Russia; Table S4: Initial data for phylogenetic analysis; Table S5: List of the RT-PCR primers used for virus and viroid detection [22,23,24,62,73,74,75,76,77,78,79,80,81,82,83,84,85,86,87,88,89,90,91,92]; Table S6: The pairwise identity matrix of the aa Pro-Pol region of GSV, GMSV, closest viruses and representatives of the genus *Fabavirus*; Table S7: The pairwise identity matrix of the aa CP region of GSV, GMSV, closest viruses and representatives of the genus *Fabavirus*; Table S8: Basic information of selected grapevine samples and viral infection; Table S9: Mapping of preprocessed reads to closest genome or contig; Table S10: Sequences of viruses and viroids uploaded to GenBank with their identifiers.

## Abbreviations

The following abbreviations are used in this manuscript:

AGVd	Australian grapevine viroid
ArMV	Arabis mosaic virus
CP	Coat protein
CTAB	Cetyltrimethylammonium bromide
CtFabV	Camphor tree fabavirus
dsDNA	Double-stranded deoxyribonucleic acid
dsRNA	Double-stranded ribonucleic acid
ELISA	Enzyme-linked immunosorbent assay
GAMaV	Grapevine asteroid mosaic-associated virus
GAPV	Grapevine alphapartitivirus
GaTLV	Grapevine-associated tymo-like virus
GFabV2	Grapevine fabavirus 2
GFkV	Grapevine fleck virus
GFLV	Grapevine fanleaf virus
GKSV	Grapevine Kizil Sapak virus
GLRaV	Grapevine leafroll-associated virus
GMSV	Grapevine Magarach secovirus
GPGV	Grapevine Pinot gris virus
GPRV	Grapevine pararetrovirus
GRBV	Grapevine red blotch virus
GRGV	Grapevine Red Globe virus
GRSPaV	Grapevine rupestris stem pitting-associated virus
GRVFV	Grapevine rupestris vein feathering virus
GSV	Grapevine secovirus
GSyV-1	Grapevine Syrah virus 1
GULV	Grapevine umbra-like virus
GVA	Grapevine virus A
GVB	Grapevine virus B
GVD	Grapevine virus D
GVF	Grapevine virus F
GV-Sat	Grapevine satellite virus
GVT	Grapevine virus T
GYSVd	Grapevine yellow speckle viroid
HSVd	Hop stunt viroid
HTS	High-throughput sequencing
ICTV	International Committee on Taxonomy of Viruses
MP	Movement protein
NCBI	National Center for Biotechnology Information
ORF	Open reading frame
RdRp	RNA-dependent RNA polymerase
RsFabV	Reaumuria songarica fabavirus
RT-PCR	Reverse transcription polymerase chain reaction
ssRNA	Single-stranded ribonucleic acid
ULV	Umbra-like virus
VCV	Vitis cryptic virus
YgSV	Yucca gloriosa secovirus
