# Supplementary material for: Virome of the Russian Grapevine Germplasm: A Final Study and Summary
Source: Plants (Basel). 2026 Sep 2;15(17):2698. doi: 10.3390/plants15172698 (PMC13567743; doi:10.3390/plants15172698)
Supplement: Supplementary file 1 [file plants-15-02698-s001.zip › Suppl. Figure Captions.pdf]

Figure S1: Phylogenetic analysis based on sequences of RNA1 of grapevine fanleaf virus (GFLV) isolates obtained in this study (red dots), other Russian isolates (green dots) and world isolates. The colored lines connect isolates assembled in the same library. Bootstrap values below 50 are not shown.

Figure S2: Phylogenetic analysis based on sequences of RNA2 of grapevine fanleaf virus (GFLV) isolates obtained in this study (red dots), other Russian isolates (green dots) and world isolates. The colored lines connect isolates assembled in the same library. Bootstrap values below 50 are not shown.

Figure S3: Phylogenetic analysis based on genome sequences of grapevine leafroll-associated virus 2 (GLRaV-2) isolates obtained in this study (red dots), other Russian isolates (green dots) and representative phylogroup members. Bootstrap values below 50 are not shown.

Figure S4: Phylogenetic analysis based on genome sequences of grapevine leafroll-associated virus 3 (GLRaV-3) isolates obtained in this study (red dots), other Russian isolates (green dots) and world isolates. Bootstrap values below 50 are not shown.

Figure S5: Phylogenetic analysis based on genome sequences of grapevine leafroll-associated virus 4 (GLRaV-4) isolates obtained in this study (red dots), other Russian isolate (green dot) and world isolates. Bootstrap values below 50 are not shown.

Figure S6: Phylogenetic analysis based on genome sequences of grapevine leafroll-associated virus 7 (GLRaV-7) isolate obtained in this study (red dot), other Russian isolates (green dots) and world isolates. Bootstrap values below 50 are not shown.

Figure S7: Phylogenetic analysis based on genome sequences of grapevine Pinot gris virus (GPGV) isolates obtained in this study (red dots), other Russian isolates (green dots) and world isolates. Bootstrap values below 50 are not shown.

Figure S8: (A) Phylogenetic analysis based on 549 bp segment of the MP/CP gene of grapevine Pinot gris virus (GPGV) isolates obtained in this study (red dots), representative virulent and latent isolates; (B) segment of the MP/CP gene used in the phylogenetic analysis; (C) multiple alignment of the terminal fragment of the MP gene. An early stop codon associated with GLMD is indicated as ter1. A stop codon prevalent in asymptomatic strains is indicated as ter2.

Figure S9: Phylogenetic analysis based on genome sequences of grapevine rupestris stem pitting-associated virus (GRSPaV) isolates obtained in this study (red dots) and representative phylogroup members. Bootstrap values below 50 are not shown.

Figure S10: Phylogenetic analysis based on genome sequences of grapevine virus T (GVT) isolates obtained in this study (red dots), other Russian isolates (green dots) and world isolates. Bootstrap values below 50 are not shown.

Figure S11: Phylogenetic analysis based on the CP gene of grapevine fleck virus (GFkV) isolates obtained in this study (red dots), other Russian isolates (green dots) and world isolates. Bootstrap values below 50 are not shown.

Figure S12: Phylogenetic analysis based on genome sequences of grapevine virus A (GVA) isolates obtained in this study (red dots), other Russian isolates (green dots) and world isolates. Bootstrap values below 50 are not shown.

Figure S13: Phylogenetic analysis based on genome sequences of grapevine virus F (GVF) isolates obtained in this study (red dots), other Russian isolates (green dots) and world isolates. Bootstrap values below 50 are not shown.

Figure S14: Phylogenetic analysis based on genome sequences of grapevine Kizil Sapak virus (GKSV) isolates obtained in this study (red dots), other Russian isolate (green dot) and world isolates. Bootstrap values below 50 are not shown.

Figure S15: Phylogenetic analysis based on genome sequences of grapevine satellite virus (GV-Sat) isolates obtained in this study (red dots), other Russian isolates (green dots) and world isolates. Bootstrap values below 50 are not shown.

Figure S16: Phylogenetic analysis based on sequences of (A) RNA1 and (B) RNA2 of Vitis cryptic virus (VCV) isolates obtained in this study (red dots), other Russian isolates (green dots) and world isolates. Bootstrap values below 50 are not shown.

Figure S17: Phylogenetic analysis based on genome sequences of grapevine yellow speckle viroid 1 (GYSVd-1) isolates obtained in this study (red dots), other Russian isolates (green dots) and representative phylogroup members. Bootstrap values below 50 are not shown.

Figure S18: Phylogenetic analysis based on genome sequences of hop stunt viroid (HSVd) isolates obtained in this study (red dots) and representative phylogroup members. Bootstrap values below 50 are not shown.

Figure S19: Phylogenetic analysis based on genome sequences of grapevine yellow speckle viroid 2 (GYSVd-2) isolate obtained in this study (red dot), other Russian isolates (green dots) and world isolates. Bootstrap values below 50 are not shown.

Figure S20: Phylogenetic analysis based on genome sequences of Australian grapevine viroid (AGVd) isolates obtained in this study (red dots), other Russian isolates (green dots) and world isolates. Bootstrap values below 50 are not shown.
